# Ishophloroglucin A, Isolated from *Ishige okamurae*, Alleviates Dexamethasone-Induced Muscle Atrophy through Muscle Protein Metabolism In Vivo

**DOI:** 10.3390/md20050280

**Published:** 2022-04-22

**Authors:** Hye-Won Yang, Seyeon Oh, Dong-Min Chung, Minyoung Seo, Shin Jae Park, You-Jin Jeon, Kyunghee Byun, BoMi Ryu

**Affiliations:** 1 Department of Marine Life Science, School of Marine Biomedical Sciences, Jeju National University, 102 Jejudaehak-ro, Jeju 63243, Korea; hyewon.yang@jejunu.ac.kr (H.-W.Y.); youjinj@jejunu.ac.kr (Y.-J.J.); 2 Functional Cellular Networks Laboratory, Department of Medicine, Graduate School, Lee Gil Ya Cancer and Diabetes Institute, Gachon University, Incheon 21999, Korea; seyeon8965@gmail.com; 3 Shinwoo Co., Ltd., Jinju 52839, Korea; jdm@shinwoocorp.com (D.-M.C.); min086@shinwoocorp.com (M.S.); sjpark@shinwoocorp.com (S.J.P.); 4 Marine Science Institute, Jeju National University, Jeju 63333, Korea; 5 Department of Anatomy & Cell Biology, College of Medicine, Gachon University, Incheon 21936, Korea

**Keywords:** muscle atrophy, Ishophloroglucin A, *Ishige okamurae*, muscle protein metabolism, muscle growth

## Abstract

The in vitro capacity of *Ishige okamurae* extract (IO) to improve impaired muscle function has been previously examined. However, the mechanism underlying IO-mediated muscle protein metabolism and the role of its component, Ishophloroglucin A (IPA), in mice with dexamethasone (Dexa)-induced muscle atrophy remains unknown. In the present study, we evaluated the effect of IO and IPA supplementation on Dexa-induced muscle atrophy by assessing muscle protein metabolism in gastrocnemius and soleus muscles of mice. IO and IPA supplementation improved the Dexa-induced decrease in muscle weight and width, leading to enhanced grip strength. In addition, IO and IPA supplementation regulated impaired protein synthesis (PI3K and Akt) or degradation (muscle-specific ubiquitin ligase muscle RING finger and atrogin-1) by modulating mRNA levels in gastrocnemius and soleus muscles. Additionally, IO and IPA upregulated mRNA levels associated with muscle growth activation (transient receptor potential vanilloid type 4 and adenosine A1 receptor) or inhibition (myostatin and sirtuin 1) in gastrocnemius and soleus muscle tissues of Dexa-induced mice. Collectively, these results suggest that IO and IO-derived IPA can regulate muscle growth through muscle protein metabolism in Dexa-induced muscle atrophy.

## 1. Introduction

Skeletal muscle is important for regulating human body metabolism. In particular, it plays a critical role in various physiological functions, maintaining the homeostasis of metabolic parameters such as basal metabolic rate, glucose uptake, and lipid utilization [1]. In addition, the maintenance of skeletal muscle mass and myofibers is regulated by a balance between the degradation and synthesis of muscle protein [2]. However, disruption of this balance can interfere with muscle fiber contraction, resulting in a less stable condition, thereby increasing susceptibility to contraction-induced damage and leading to muscle atrophy [3].

Muscle atrophy, attributed to increased myofibrillar protein breakdown and decreased synthesis, is the loss of skeletal muscle mass during various conditions, including fasting, disuse, injury, side effects associated with pharmaceutical drug therapy, and aging [4]. Abnormal weakness in muscle mass, muscle strength, and function owing to muscle atrophy can be associated with poor prognosis and resistance to pharmaceutical treatment in cancer patients, resulting in increased morbidity and mortality [5]. Moreover, muscle atrophy decreases the quality of life due to frequent falls and reduces the ability to walk [6]. Therefore, preventing muscle atrophy and improving muscle strength by employing appropriate therapeutic agents is an attractive approach.

Glucocorticoids are widely used for treating various diseases and are indispensable for regulating physiological processes [7]. In addition, glucocorticoids play an important role in mediating muscle protein degradation and the upregulation of the ubiquitin-proteasome pathway in skeletal muscles [8]. Although glucocorticoids afford considerable benefits, excessive or prolonged use can cause muscle atrophy by inhibiting amino acid transport into the muscle while suppressing muscle protein synthesis and stimulating muscle protein degradation [9,10]. A previous study reported that dexamethasone (Dexa), a synthetic glucocorticoid, can cause muscle atrophy via the induction of protein degradation and the suppression of typical protein synthesis [11]. In particular, a high dose of Dexa increases the expression of muscle-atrophy-related muscle-specific ubiquitin ligases, such as muscle RING finger 1 (MuRF1) and atrogin-1 [12,13].

In addition, Dexa-induced muscle atrophy has been established as an in vivo muscle atrophy model [14,15]. Rodents, particularly mice and rats, are frequently used as animal models to examine specific phenotypes of muscles, using a more uniform distribution among different muscles [16]. In addition, the soleus and gastrocnemius of mice are mainly composed of two muscle fibers, fast- and slow-twitch muscle fibers, respectively [16,17,18]. In previous studies, soleus and gastrocnemius muscles have been used to investigate the molecular mechanism of Dexa-induced muscle atrophy in mice [7,15,19].

*Ishige okamurae* is an edible brown alga composed of isophloroglucin A (IPA) and diphlorethohydroxycarmalol (DPHC). *Ishige okamurae* extract (IO) has been reported to activate skeletal muscle cell proliferation without inducing cytotoxicity in C2C12 myoblasts [20]. In addition, IO can potentially inhibit tumor necrosis factor (TNF)-α in muscle-degradation-induced inflammatory myopathy [21]. However, the underlying mechanism of action of IPA, which is the equivalent content of IPA in IO, on muscle function in Dexa-induced muscle atrophy remains unknown.

Therefore, in the present study, we examined the effects of IO and its component IPA on muscle atrophy using a Dexa-induced mouse model. In addition, we assessed the regulation of muscle atrophy in the soleus and gastrocnemius muscles of mice to clarify muscle protein metabolism. Based on our findings, IO and IPA may improve muscle function in mice with Dexa-induced muscle atrophy, and IO-derived IPA could be developed as a prospective functional supplement for enhancing muscle atrophy.

## 2. Results

### 2.1. Effect of IO and IO-Derived IPA Supplementation on Physiological Characteristics in the Dexa-Induced Muscle Atrophy Model

**Figure 1:**
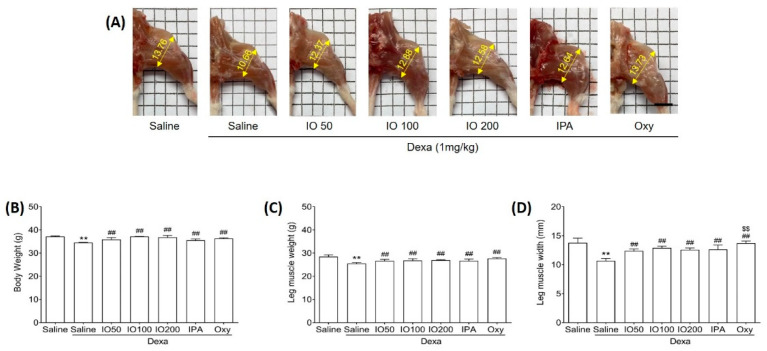
Effect of IO and IPA supplementation on physiological characteristics in the dexamethasone (Dexa)-induced muscle atrophy mouse model. Mice were treated with Dexa (1 mg/kg) by subcutaneous injection for 10 days and co-administered IO (50, 100, or 200 mg/kg), IPA (2 mg/kg), and Oxy (50 mg/kg) for 28 days. After 38 days, (**A**) morphological images in the calf muscle, (**B**) body weight, (**C**) leg muscle weight, and (**D**) leg muscle width were measured in all mice groups. Each group was examined in *n* = 7 mice. Data are expressed as a mean ± standard deviation (SD). ** *p* < 0.01 vs. saline; ## *p* < 0.01 vs. Dexa/saline; $$ *p* < 0.01 vs. Dexa/IO100. IO, *Ishige okamurae* extract; IPA, Ishophloroglucin A; Oxy, oxymetholone.

To determine whether IO and IPA can impact the morphological properties of muscles, we evaluated muscle weight and size in mice with Dexa-induced muscle atrophy. The dosage of IPA supplementation (2 mg/kg) was equivalent to the content present in IO supplementation (100 mg/kg). Mice were treated with a subcutaneous injection of Dexa (1 mg/kg) for 10 days to induce muscle atrophy. In addition to Dexa (1 mg/kg) injection, we administered IO (50, 100, or 200 mg/kg), IPA (2 mg/kg), and oxymetholone (Oxy; 50 mg/kg), which was used as a positive control to assess muscle properties in muscle atrophy [22]. After 10 days, Dexa/saline demonstrated markedly distinct morphological muscle images when compared with saline only, as shown in Figure 1A. A previous study reported that Dexa treatment in mice can induce morphological changes, as well as decrease muscle mass [23]. However, treatment with IO, IPA, and Oxy could alleviate the changes observed in morphological muscle images. Additionally, we measured the body weights of the mice before the start of the experiment (Figure S1A), after oral administration of IO and IPA for 28 days before Dexa injection (Figure S1B), and at the end of the experiment (Figure 1B). After oral administration of IO and IPA for 4 weeks, it was confirmed that there was no difference in the weight of the mice compared to saline (Figure S1A,B). However, Dexa significantly decreased body weights compared with saline, while no significant differences in Dexa/IO 100 and Dexa/IO 200 were observed (Figure 1B). Moreover, decreased leg muscle weights by Dexa were significantly enhanced following IO, IPA, and Oxy supplementation (Figure 1C). The mean leg muscle width in Dexa/IO was 12.88 mm when compared with 10.66 mm in Dexa/saline, indicating that the leg muscle width was significantly increased 1.2-fold (Figure 1D). Overall, these results demonstrate that IPA and IO-derived IPA could ameliorate the morphological properties of muscles in Dexa/saline. Furthermore, we evaluated the improvement in muscle function during muscle atrophy using gastrocnemius and soleus muscle tissues.

### 2.2. Effect of IO and IO-Derived IPA Supplementation on Grip Strength in Dexa-Induced Muscle Atrophy Model

**Figure 2:**
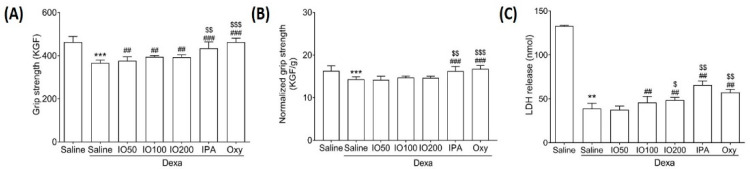
Effect of IO and IPA supplementation on calf muscle strength in the Dexa-induced muscle atrophy mouse model. (**A**) Muscle strength was measured using a grip strength meter. (**B**) Grip strength was normalized for muscle weight. (**C**) LDH release was measured using an ELISA kit. Each group was examined in *n* = 7 mice. Data are expressed as a mean ± standard deviation (SD). ** *p* < 0.01 vs. saline, *** *p* < 0.001 vs. saline; ## *p* < 0.01 vs. Dexa/saline and ### *p* < 0.001 vs. Dexa/saline; $ *p* < 0.05 vs. Dexa/IO100, $$ *p* < 0.01 vs. Dexa/IO100, and $$$ *p* < 0.001 vs. Dexa/IO100. Dexa, dexamethasone; IO, *Ishige okamurae* extract; IPA, Ishophloroglucin A; Oxy, oxymetholone.

Muscle atrophy might be accompanied by alterations in muscle size and grip strength [24]. To evaluate the effect of IO and IPA on grip strength, we measured the exercise capacity in the Dexa-induced muscle atrophy model. As shown in Figure 2A, the grip strength was significantly decreased in Dexa/saline when compared with saline; however, IO, IPA, and Oxy supplementation markedly preserved the reduced strength. In addition, IPA supplementation significantly increased grip strength when compared with Dexa/IO100. Moreover, grip strength was assessed by normalization for muscle weight (Figure 2B). As shown in Figure 2C, lactate dehydrogenase (LDH) release was markedly reduced in Dexa/saline. Conversely, the decreased LDH levels were significantly increased following IO, IPA, and Oxy supplementation. These findings suggest that IPA and IO-derived IPA could improve muscle function in the Dexa-induced muscle atrophy model.

### 2.3. Effect of IO and IO-Derived IPA Supplementation on Muscle Mass in Gastrocnemius and Soleus Muscle Tissue of Mice

**Figure 3:**
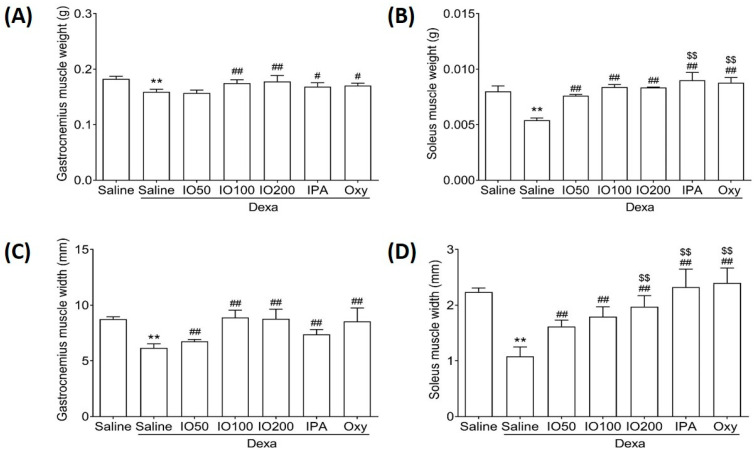
Effect of IO and IPA supplementation on the muscle mass of gastrocnemius and soleus muscle tissues in mice with muscle atrophy. The weight of the (**A**) gastrocnemius muscle or (**B**) soleus muscle and the width of the (**C**) gastrocnemius muscle or (**D**) soleus muscle were measured using an automatic electronic balance machine in all mice groups. Each group was examined in *n* = 7 mice. Data are expressed as a mean ± standard deviation (SD). ** *p* < 0.01 vs. saline; # *p* < 0.05 vs. Dexa/saline, ## *p* < 0.01 vs. Dexa/saline; $$ *p* < 0.01 vs. Dexa/IO100. Dexa, dexamethasone; IO, *Ishige okamurae* extract; IPA, Ishophloroglucin A; Oxy, oxymetholone.

Muscle recovery can help maintain the balance between fast and slow contraction in gastrocnemius and soleus muscles [19]. Accordingly, we examined whether IO and IPA supplementation could impact muscle mass in gastrocnemius and soleus muscle tissues. As shown in Figure 3A,B, the weight of gastrocnemius and soleus was significantly decreased in Dexa/saline compared with saline; however, IO, IPA, and Oxy supplementation could recover the decreased weight. In addition, the widths of the gastrocnemius and soleus were significantly increased in Dexa/IO or Dexa/IPA compared with Dexa/saline (Figure 3C,D). Interestingly, the weight and width of the soleus muscle were significantly elevated in Dexa/IPA compared with Dexa/IO100. H&E staining analysis of the gastrocnemius and soleus muscle tissues of the saline group revealed that the muscle fibers were closely wrapped by the perimysium to form muscle bundles (Figure S2). By contrast, in the Dexa/saline group, the spacing between the muscle fibers was different. The perimysium plays a role in the lateral transmission of contractile forces [25,26]. These results indicate that the IO- and IPA-induced increase in muscle weight and width could maintain balanced muscle recovery during muscle atrophy.

### 2.4. Effect of IO and IO-Derived IPA Supplementation on Protein Synthesis and Degradation in Skeletal Muscle Tissues of Mice

**Figure 4:**
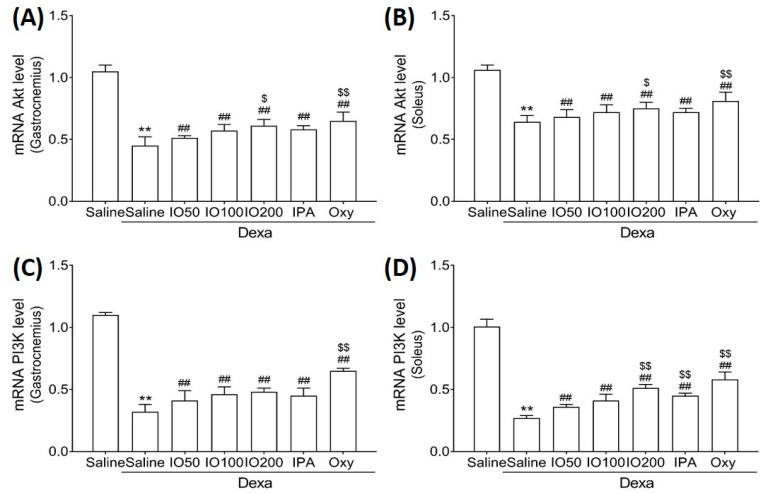
Effect of IO and IPA supplementation on protein synthesis in the gastrocnemius and soleus muscle tissues of mice with muscle atrophy. mRNA expression levels of Akt in (**A**) gastrocnemius muscle or (**B**) soleus muscle and mRNA expression levels of PI3K in (**C**) gastrocnemius muscle or (**D**) soleus muscle were measured with qRT-PCR. Each group was examined in *n* = 7 mice. Data are expressed as a mean ± standard deviation (SD). ** *p* < 0.01 vs. saline; ## *p* < 0.01 vs. Dexa/saline; $ *p* < 0.05 vs. Dexa/IO100, $$ *p* < 0.01 vs. Dexa/IO 100. IO, *Ishige okamurae* extract; IPA, Ishophloroglucin A; Oxy, oxymetholone.

Protein synthesis and degradation can regulate processes that increase or decrease muscle mass [27]. Accordingly, we examined whether IO and IPA regulate protein synthesis and degradation in the gastrocnemius and soleus muscle tissues of mice with muscle atrophy and measured mRNA levels using quantitative reverse-transcription polymerase chain reaction (qRT-PCR). As shown in Figure 4A,B, qRT-PCR analyses revealed that the Akt mRNA level in the soleus muscle tissue was significantly decreased in Dexa/saline; however, this level was significantly increased in Dexa/IO or Dexa/IPA when compared with Dexa/saline. In addition, the PI3K mRNA level was significantly decreased in Dexa/saline; however, this level was partly rescued in Dexa/IO or Dexa/IPA (Figure 4C,D). Interestingly, PI3K mRNA levels were significantly elevated in the soleus muscle tissues in the Dexa/IPA group compared to those in Dexa/IO100. IO and IPA supplementation activated the suppressed PI3K/Akt signaling pathway, indicating that IO and IPA can stimulate protein synthesis during muscle atrophy.

**Figure 5:**
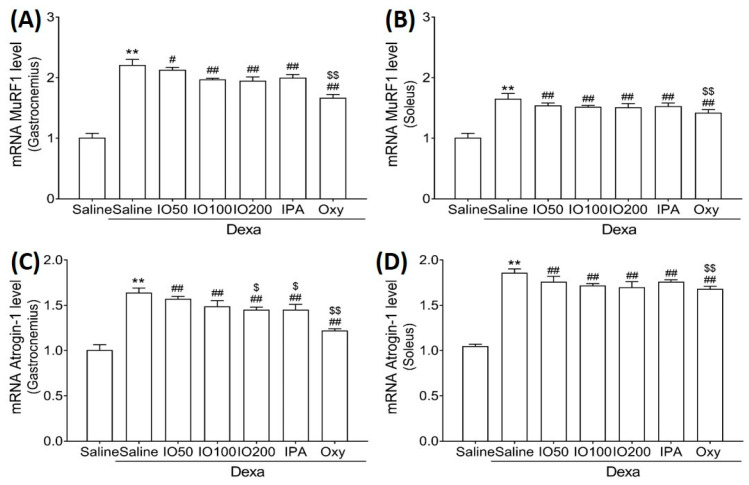
Effect of IO and IPA supplementation on protein degradation in the gastrocnemius and soleus muscle tissues of mice with muscle atrophy. mRNA expression levels of MuRF in (**A**) gastrocnemius muscle or (**B**) soleus muscle and mRNA expression levels of Atrogin-1 in (**C**) gastrocnemius muscle or (**D**) soleus muscle were measured with qRT-PCR. Each group was examined in *n* = 7 mice. Data are expressed as a mean ± standard deviation (SD). ** *p* < 0.01 vs. saline; # *p* < 0.05 vs. Dexa/saline, ## *p* < 0.01 vs. Dexa/saline; $ *p* < 0.05 vs. Dexa/IO 100, $$ *p* < 0.01 vs. Dexa/IO 100. IO, *Ishige okamurae* extract; IPA, Ishophloroglucin A; Oxy, oxymetholone.

Moreover, we evaluated the mRNA levels of muscle-specific ubiquitin ligase muscle RING finger 1 (MuRF1) and atrogin-1, specific markers of protein degradation associated with muscle atrophy. In Figure 5A,B, the mRNA level of MuRF1 in skeletal muscles was significantly increased in Dexa/saline; however, this level was significantly decreased in Dexa/IO or Dexa/IPA compared with Dexa/saline. In addition, the mRNA levels of atrogin-1 were increased in Dexa/saline; this effect was observed following the co-administration of Dexa/IO or Dexa/IPA compared with Dexa/saline (Figure 5C,D). IO and IPA supplementation suppressed the activated molecular mechanism mediated by MuRF1 and atrognin-1, indicating that IO and IPA can suppress protein degradation during muscle atrophy. These findings suggest that IO and IPA administration could improve muscle function by regulating protein synthesis and degradation pathways in muscle atrophy.

### 2.5. Effect of IO and IO-Derived IPA Supplementation on Muscle Growth Activation and Inhibition in Skeletal Muscle Tissues of Mice

**Figure 6:**
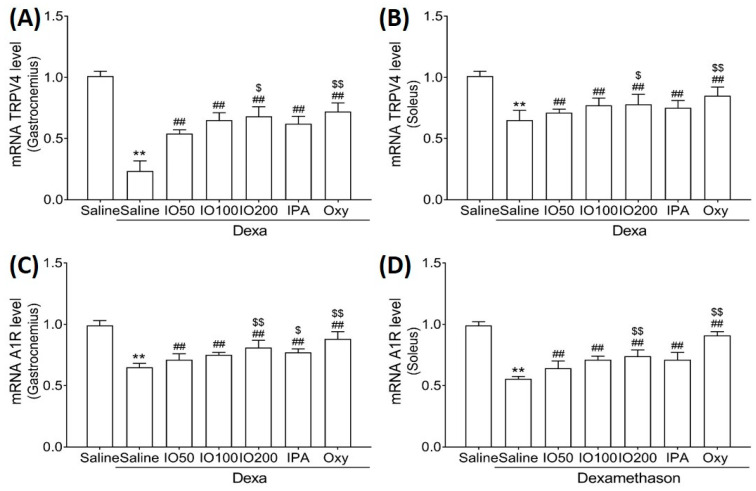
Effect of IO and IPA supplementation on muscle growth activation in gastrocnemius and soleus muscle tissues of mice with muscle atrophy. mRNA expression levels of TRPV4 in (**A**) gastrocnemius muscle or (**B**) soleus muscle and mRNA expression levels of A1R in (**C**) gastrocnemius muscle or (**D**) soleus muscle were measured with qRT-PCR. Each group was examined in *n* = 7 mice. ** *p* < 0.01 vs. saline; ## *p* < 0.01 vs. Dexa/saline; $ *p* < 0.05 vs. Dexa/IO 100, $$ *p* < 0.01 vs. Dexa/IO 100. IO, *Ishige okamurae* extract; IPA, Ishophloroglucin A; Oxy, oxymetholone.

To clarify the molecular mechanisms underlying muscle growth, the regulatory effect of IO and IPA supplementation on activation and inhibitory pathways was determined using qRT-PCR. As shown in Figure 6A,B, qRT-PCR analyses revealed that the skeletal muscle mRNA level of transient receptor potential vanilloid type 4 (TRPV4) was significantly decreased in Dexa/saline; however, this level was significantly increased in Dexa/IO or Dexa/IPA compared with that in Dexa/saline. In addition, the mRNA levels of A1R were indeed decreased upon Dexa/saline treatment; the co-administration of Dexa and IPA enhanced A1R mRNA levels with a statistical significance of *p* < 0.01 (Figure 6C,D). In particular, the mRNA levels of TRPV4 and A1R in gastrocnemius muscle markedly differed between Dexa/IPA and Dexa/IO100. These results indicate that IPA could activate muscle growth during muscle atrophy.

**Figure 7:**
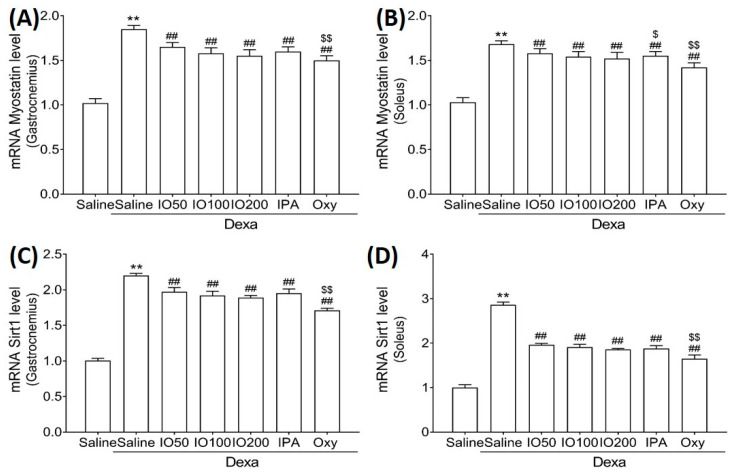
Effect of IO and IPA supplementation on muscle growth inhibition in the gastrocnemius and soleus muscle tissues of mice with muscle atrophy. mRNA expression levels of Myostatin in (**A**) gastrocnemius muscle or (**B**) soleus muscle and mRNA expression levels of Sirt1 in (**C**) gastrocnemius muscle or (**D**) soleus muscle were measured with qRT-PCR. Each group was examined in *n* = 7 mice. ** *p* < 0.01 vs. saline; ## *p* < 0.01 vs. Dexa/saline; $ *p* < 0.05 vs. Dexa/IO 100, $$ *p* < 0.01 vs. Dexa/IO 100. IO, *Ishige okamurae* extract; IPA, Ishophloroglucin A; Oxy, oxymetholone.

Moreover, the mRNA levels of myostatin and sirtuin 1 (Sirt1), associated with muscle growth differentiation factors, were significantly elevated in Dexa/saline. However, the Dexa/IO or Dexa/IPA groups showed significantly reduced mRNA levels of myostatin (Figure 7A,B) and Sirt1 (Figure 7C,D) compared to Dexa/saline. These findings suggest that IO and IPA regulated the activation and inhibition pathways, indicating that the administration of IO and IPA could improve muscle growth during muscle atrophy.

## 3. Discussion

Previous studies have reported the various biological activities mediated by IO, including anti-obesity, anti-diabetes, and anti-inflammatory properties [28,29]. A previous study showed that IO downregulated pro-inflammatory cytokines and muscle atrophy proteins against TNF-α, thereby demonstrating its potential as a TNF-α inhibitor for inflammatory myopathy [21]. In addition, IO improved the proliferation of C2C12 myoblasts [20]. However, the in vivo ability of IO and IPA, as a component of IO, to improve muscle function in Dexa-induced muscle atrophy remains unknown. In the present study, our findings revealed that IO and an equivalent amount of IPA in IO prevented Dexa-induced muscle atrophy by facilitating a balance between protein synthesis and degradation.

Dexa has been previously employed in both in vitro and in vivo skeletal muscle atrophy models [14,30]. High-dose Dexa can induce skeletal muscle atrophy by reducing muscle tension, which is related to decreased protein synthesis and increased protein degradation, resulting in a shortening of muscle fibers and a loss of overall muscle mass [31,32,33]. Dexa-induced muscle atrophy had little or no effect on slow-twitch fibers (type I), whereas it induced a selective loss of fast-twitch fibers (type II) [34]. In addition, previous studies have shown that the loss of muscle mass can contribute to the loss of muscle strength [15,35]. LDH release has been used as a marker of cell damage in muscle myotubes [36]. IO and IPA significantly increased body weight, muscle weight, LDH release, and leg muscle width and strength in mice with muscle atrophy. This confirmed that there was a difference in weight after Dexa injection, as reported in other studies [32,37]. These results suggest a protective action of IO-derived IPA against muscle dysfunction in Dexa-induced muscle atrophy.

During activities such as walking and exercise, calf muscles allow for forward movement by regulating two main muscles, i.e., the gastrocnemius and the soleus muscle [38]. In addition, the gastrocnemius and soleus muscles help maintain the balance in muscle protein metabolism. In previous studies, gastrocnemius and soleus muscle tissues were employed to evaluate the regulation of muscle protein metabolism in Dexa-induced muscle atrophy [15,39]. Herein, we observed that the decreased muscle mass of the gastrocnemius and soleus muscles was notably improved in mice with muscle atrophy following IPA supplementation, probably due to the regulation of protein synthesis and degradation. In particular, muscle-specific ubiquitin ligases such as MuRF1 and Atrogin-1 are related to muscle atrophy induced by a high dose of Dexa [12,13]. In addition, the activation of the PI3K/Akt signaling pathway in skeletal muscle is associated with protein synthesis and degradation, leading to an inhibition of the ubiquitin-proteasome-dependent proteolytic pathway [40,41,42]. The mechanism underlying the IO- and IPA-mediated improvement in muscle mass was associated with PI3K, Akt, MuRF1, and atrogin-1, facilitating the maintenance of balanced muscle protein metabolism.

Muscle atrophy is characterized by an imbalance between protein synthesis and degradation [30,43]. The regulation of muscle protein synthesis and degradation can be correlated with the metabolic basis of skeletal muscle growth [44]. Reportedly, the mRNA levels of TRPV4 and A1R are associated with muscle growth and the physiological functions of skeletal muscle [37]. In this study, mRNA levels in the Dexa group showed a decrease in TRPV4 and A1R (involved in muscle growth) and an increase in myostatin (a potent negative regulator of muscle growth) and SIRT1 (a representative inhibitor of muscle regeneration) [37]. Myostatin, a muscle growth and differentiation factor, is a negative regulator of muscle growth [45]. Moreover, SIRT1 activation can downregulate the myostatin signaling pathway [46]. A previous study reported that myostatin plays a role in regulating myoblast proliferation via SIRT1 [47]. IO and IPA upregulated mRNA levels related to muscle growth (TRPV4 and A1R) while downregulating mRNA levels associated with muscle growth inhibition (myostatin and SIRT1). This indicates that IO-derived IPA improved muscle growth by inhibiting the myostatin signaling pathway.

## 4. Materials and Methods

### 4.1. Isolation IPA from IO

IO (Lot No. SW9E22SA) extracted in 50% ethanol was provided by Shinwoo Co., Ltd. (Gyeonggi-do, Korea). In the present study, IO contained 2.36% IPA and 2.36% DPHC (Figure S3), determined by high-pressure liquid chromatography analysis, as previously described [48].

### 4.2. Dexamethasone (Dexa)-Induced Muscle Atrophy Model

Male CrljOri:CD1 (ICR) mice (9-week-old, 33 ± 3 g) were purchased from OrientBio Inc. (Seongnam, Korea). Female mice were excluded because hormones such as estrogen mediate exercise capacity in female mice [49], which could have affected the results in this study. The animals were housed under controlled environmental conditions (23 ± 2 °C and 50 ± 5% humidity with 12 h light/dark cycle) with ad libitum access to water and a regular rodent chow diet. After acclimatization for one week, all the mice were randomly divided into seven groups, with seven mice per group, as follows: (1) saline: mice were administered saline; (2) Dexa/saline: mice were administered saline and dexamethasone (1 mg/kg/day); (3) Dexa/IO50: mice were administered 50 mg/kg/day IO and Dexa; (4) Dexa/IO100: mice were administered 100 mg/kg/day IO and Dexa; (5) Dexa/IO200: mice were administered 200 mg/kg/day IO and Dexa; (6) Dexa/IPA: mice were administered 2 mg/kg/day IPA and Dexa; (7) Dexa/Oxy: mice were administered 50 mg/kg/day Oxy and Dexa. IO and IPA were dissolved in saline, and the control group was administered the same amount of saline water. IO and IPA were orally administered once daily for 28 days, prior to Dexa injection, and maintained throughout the experimental period of 38 days. Herein, the muscle atrophy model employed induced muscle atrophy following the subcutaneous injection of Dexa (1 mg/kg) once daily for 10 days [32,37]. The body weight, fat mass, lean mass, grip strength, muscle thickness, and weight of each mouse were measured for 38 days. Subsequently, the gastrocnemius and soleus muscles were harvested in accordance with the ethical principles of the Institutional Animal Care and Use Committee of Gachon University (approval no. LCDI-2020-0003) [50].

### 4.3. Measurement of Grip Strength

A grip strength meter (JD-A-22, JEUNGDO BIO& PLANT Co., LTD., Seoul, Korea) was used to measure the grip strength of the experimental mice. The grip strength meter digitally displays the maximum force. After placing the mouse on the meter bar, the tail was carefully pulled, ten measurements were performed at 1 min intervals, and the average was used.

### 4.4. Measurement of LDH Release

LDH assays (ab102526, Abcam, Cambridge, UK) in the serum of each group were determined using the appropriate kit, in accordance with the manufacturer’s instructions.

### 4.5. Measurement of Leg Muscle Weight

We weighed individual leg muscle masses in grams (absolute wet weight) using an automatic electronic balance machine and then calculated relative weight (% of body weight) to minimize differences among individual body weights.

### 4.6. RNA Extraction and qRT-PCR

Briefly, frozen muscle tissue was lysed with 500 μL of RNiso (Takara, Shiga, Japan) using a homogenizer. After mixing with chloroform, samples were centrifuged at 12,000× *g* for 15 min at 4 °C. The aqueous phase was collected in cleaned tubes, mixed with isopropanol (amount equivalent to the aqueous layer), and then centrifuged at 12,000× *g* for 15 min at 4 °C. Isolated RNA samples were washed with 75% ethanol and dried. Then, the RNA pellet was dissolved with diethyl pyrocarbonate-treated water. cDNA was synthesized using a PrimeScript First Strand cDNA Synthesis Kit (Takara, Shiga, Japan). qRT-PCR was performed using the CFX 384 Touch™ Real-Time PCR Detection System (Bio-Rad Laboratories, Irvine, CA, USA). The reaction efficiency and cycle threshold values were identified with the CFX Manager™ software (Bio-Rad Laboratories, Irvine, CA, USA). Actb was used as the internal control, and primer sequences for the target genes are listed in Supplementary Table S1.

### 4.7. Statistical Analysis

Results are expressed as the mean ± standard deviation (SD) and analyzed using SPSS software (verstion 22, IBM Corp., Armonk, NY, USA). Significant differences between the groups were compared using the Kruskal–Wallis and Mann–Whitney U post-hoc tests. The experiments were performed in triplicate with seven animals per group. Statistical significance was established as *p* < 0.05. (*) indicates a significant difference from the saline, and (#) represents a significant difference compared with Dexa/saline.

## 5. Conclusions

In conclusion, our findings are as follows: the administration of IO and IPA (1) ameliorated the morphological properties of the examined skeletal muscles, (2) regulated protein metabolism between protein synthesis and degradation, and (3) improved muscle growth by regulating the activation/inhibition of muscle regeneration, thereby improving the imbalance in muscle protein metabolism caused by muscle atrophy. IO and IPA could be potential therapeutic or functional food candidates for preventing the muscle atrophy-induced imbalance in muscle protein metabolism. Further studies are needed to clarify the correlation between metabolic pathways and the administration of IPA in Dexa-induced muscle atrophy.

## Data Availability

Not applicable.

## Supplementary Materials

The following supporting information can be downloaded at: https://www.mdpi.com/article/10.3390/md20050280/s1. Table S1: List of primers for qRT-PCR; Figure S1: Body weight of mice at 0 days (Figure S1A) and 28 days (Figure S1B); Figure S2: H&E staining of gastrocnemius and soleus muscle fibers; Figure S3: HPLC chromatogram of IPA and DPHC from IO.
