# Supplementary material for: Ishophloroglucin A, Isolated from Ishige okamurae, Alleviates Dexamethasone-Induced Muscle Atrophy through Muscle Protein Metabolism In Vivo
Source: Mar Drugs. 2022 Apr 22;20(5):280. doi: 10.3390/md20050280 (PMC9147101; doi:10.3390/md20050280)
Supplement: Supplementary file 1 [file marinedrugs-20-00280-s001.zip › marinedrugs-1659704-supplementary.pdf]

Table S1. List of primer for qRT-PCR

Gene		Primers
Akt	Forward	5’-CCT TTA TTG GCT ACA AGG AAC G-3’
	Reverse	5’-GTG CCA CTG AGA AGT TGT TGA G-3’
PI3k	Forward	5’-AGC TGA GTA CCG AGA GAT CGA C-3’
	Reverse	5’-TCA GCC ACA TCA AGT ATT GGT C-3’
MuRF1	Forward	5'-ATC TAG CCT GAT TCC TGA TGG A-3’
	Reverse	5'-ACC ACA GGC TTG GTA AAC ATC T-3’
Atrogin-1	Forward	5'-GAC ATT CAG AAC AGC AAA ACC A-3’
	Reverse	5'-GCT CCT TCG TAC TTC CTT TGT G-3’
Trpv4	Forward	5’-CCCATCCTCAAAGTCTTCAATC -3’
	Reverse	5’- GGTCAACAAGAAGGAGAGCAGT-3’
A1r	Forward	5’-GAAGATTTGGAACGACCACTTC -3’
	Reverse	5’-AGTCATCAGCTTTCTCCTCTGG -3’
Myostatin	Forward	5’- TTTACCTGTTCATGCTGATTGC-3’
	Reverse	5’- CATTACACAGCCCCTCTTTTTC-3’
Sirt1	Forward	5’-TGTGTGGAAGAAAAACCACAAG -3’
	Reverse	5’- ACCAACAGCCTTAAAATCTGGA -3’

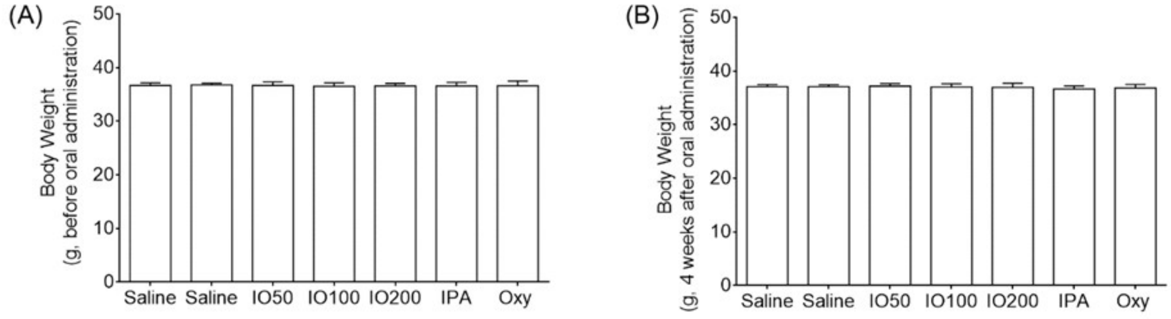

Figure S1. The body weight of mouse at 0 days (Figure S1A) and 28 days (Figure S1B).

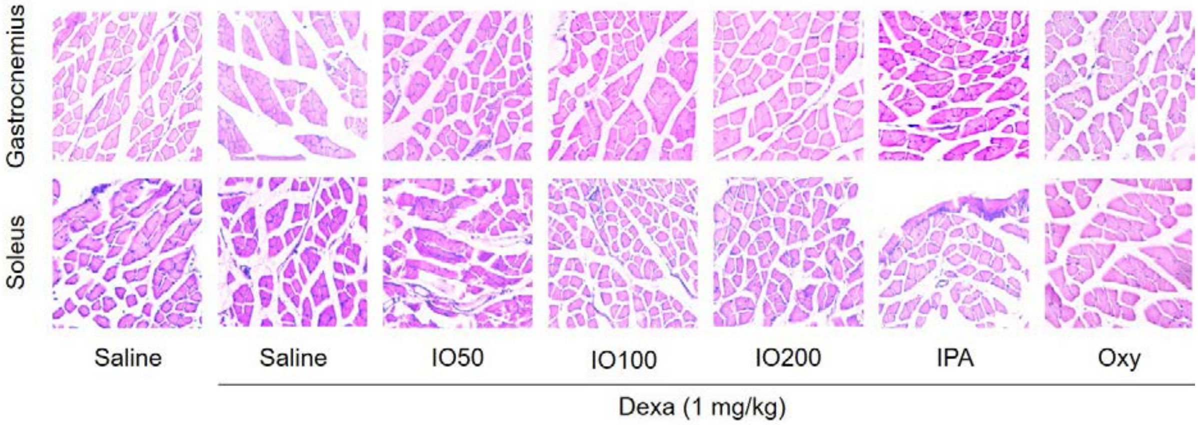

Figure S2. H&E staining of gastrocnemius and soleus muscle fibers

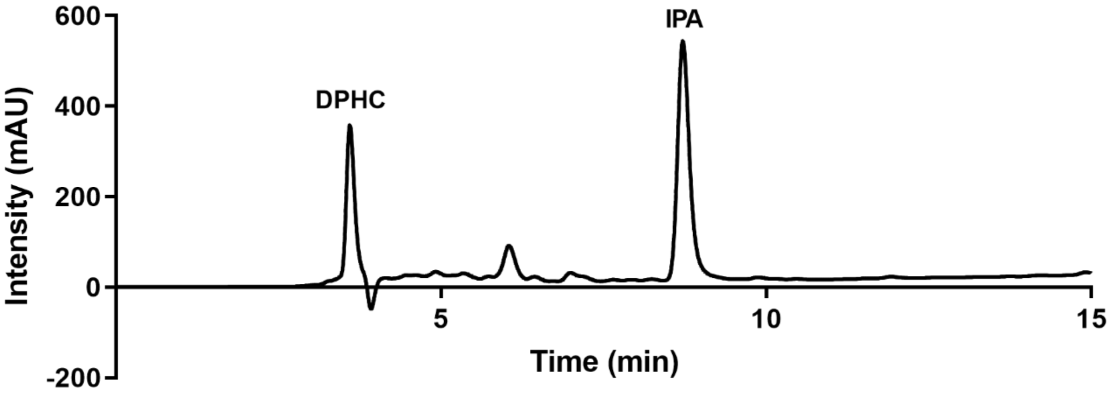

Figure S3. HPLC chromatogram of IPA and DPHC from IO.
